# Activated Carbon Fibers “Thickly Overgrown” by Ag Nanohair Through Self-Assembly and Rapid Thermal Annealing

**DOI:** 10.1186/s11671-017-2344-x

**Published:** 2017-11-09

**Authors:** Xuefeng Yan, Sijun Xu, Qiang Wang, Xuerong Fan

**Affiliations:** 1 Key Laboratory of Eco-Textiles, Ministry of Education, Jiangnan University, Wuxi, 214122 People’s Republic of China; 2 School of Textile and Clothing, Nantong University, Nantong, 226019 People’s Republic of China

**Keywords:** Ag nanohair, Active carbon fibers, Self-assembly, Rapid thermal annealing

## Abstract

Anisotropic nanomaterial-modified carbon fibers attract increasing attention because of their superior properties over traditional ones. In this study, activated carbon fibers (ACFs) “thickly overgrown” by Ag nanohair were prepared through self-assembly and rapid thermal annealing. Viscose fibers with well-dispersed silver nanoparticles (AgNPs) on surfaces were first prepared through self-assembly of hyperbranched poly(amino-amine) (HBPAA)-capped AgNPs on viscose surfaces. HBPAA endowed the AgNP surfaces with negative charges and abundant amino groups, allowing AgNPs to monodispersively self-assemble to fiber surfaces. Ag nanohair-grown ACFs were prepared by sequential pre-oxidation and carbonization. Because the carbonization furnace was open-ended, ACFs are immediately transferrable to the outside of the furnace. Therefore, the Ag liquid adsorbed by ACF pores squeezed out to form Ag nanowires through thermal contraction. FESEM characterization indicated that Ag nanohairs stood on ACF surface and grew from ACF caps. XPS and XRD characterization showed that Ag successfully assembled to fiber surfaces and retained its metallic state even after high-temperature carbonization. TG analysis suggested that Ag nanohair-grown ACFs maintained their excellent thermal stabilities. Finally, the fabricated ACFs showed excellent and durable antibacterial activities, and the developed method may provide a potential strategy for preparing metal nanowire-grown ACFs.

## Background

Carbon fibers (CFs) can be defined as fibers that consist of at least 92% carbon by weight and are prepared from polymeric precursors, such as polyacrylonitrile (PAN), pitch, cellulose, lignin, and polyethylene [1, 2]. PAN was first used as a precursor for the preparation of CFs and still remains as an important starting material. With the development of the manufacturing industry, the demand for CFs has greatly increased because of their outstanding performances such as high tensile strengths, low densities, high moduli, excellent chemical and thermal stability, and/or strong adsorption ability for various inorganic and organic materials. However, the production cost of CFs is one major obstacle in large-scale applications. Biological materials such as biopolymers or polymers from biogenic sources are especially interesting sources for CFs and are inexpensive [1].

Viscose fibers (VFs) are typical regenerated cellulose fibers frequently used for the preparation of active carbon fibers (ACFs). Cellulose-based ACFs possess much weaker mechanical properties than CFs although the adsorption ability of the former is much stronger than the latter [3]. The specific surface area of ACFs is up to 1000–1500 m^2^/g, with millions of 1–4-nm micropores dispersed on the fiber surface. Therefore, ACFs show superior adsorption ability to activated carbon, making them potentially applicable in wastewater treatment, air purification, individual protection, and so on [4, 5]. Nowadays, nanoscience and technology has made a remarkable headway. The integration of nanomaterials and carbon materials has become a popular research topic because of their outstanding properties. The fabricated composites not only inherit their respective advantages but also obtain new advanced functions under synergistic effects [6, 7]. For example, Ding et al. prepared Ag nanoparticle (AgNP)-decorated CFs by simple dipping, and the composite CFs showed fourfold higher photocatalytic activity than pure AgNPs during conversion of CO_2_ to CH_3_OH, which primarily resulted from higher CO_2_ adsorption and more efficient electron transfer from AgNPs to CO_2_ [1]. Wan et al. synthesized highly dispersed CoSe_2_ nanoparticles on three-dimensional nanonet-like CFs by electrostatic spinning, and the electrocatalyst product possessed highly active, efficient, and stable properties for hydrogen evolution in acidic media [8]. However, current nanomaterials, especially inorganic nanomaterials, are usually spherical. With the increasingly high requirement on the performances of nanomaterial/CF composites, modification of CFs with anisotropic nanomaterials such as nanowires, nanosheets, and nano-quantum dots have become a focus because of their certain superior properties over nanoparticles [9].

In this study, we designed ACFs “thickly overgrown” by Ag nanohair through self-assembly and rapid thermal annealing. Hyperbranched poly(amino-amine) (HBPAA)-modified AgNPs were synthesized by hydrothermal reduction on the HBPAA template. With HBPAA serving as a “molecular glue,” positively charged AgNPs uniformly self-assembled to the fiber surfaces through intermolecular electrostatic and hydrogen-bonding interactions between HBPAA and viscose cellulose. Ag nanohair-grown ACFs were prepared by pre-oxidation and carbonization of AgNP-coated VFs. To successfully grow Ag nanohairs on ACFs, an open-ended carbonization furnace sealed by high-temperature flames in the entrance and exit was chosen. Therefore, ACFs could rapidly cool down upon leaving the furnace, triggering the fast cold contraction of pores. Ag liquid would be squeezed out and cooled down to form Ag nanowires.

## Methods

### Preparation of Ag Nanohair-Grown ACFs

Molecular-mediated self-assembly technology was applied to guide the AgNPs into the VF surface, forming a monodispersive coating. Briefly, HBPAA-capped AgNPs were firstly synthesized as described in our previous study [10]. Then, self-assembly of AgNPs on VFs were conducted by impregnation with 2 g VFs in a solution of HBPAA-capped AgNPs (4000 mg/L) at 98 °C for 3 h. AgNP-coated VFs were dried in an oven and stored in the dark.

**Fig. 1:**
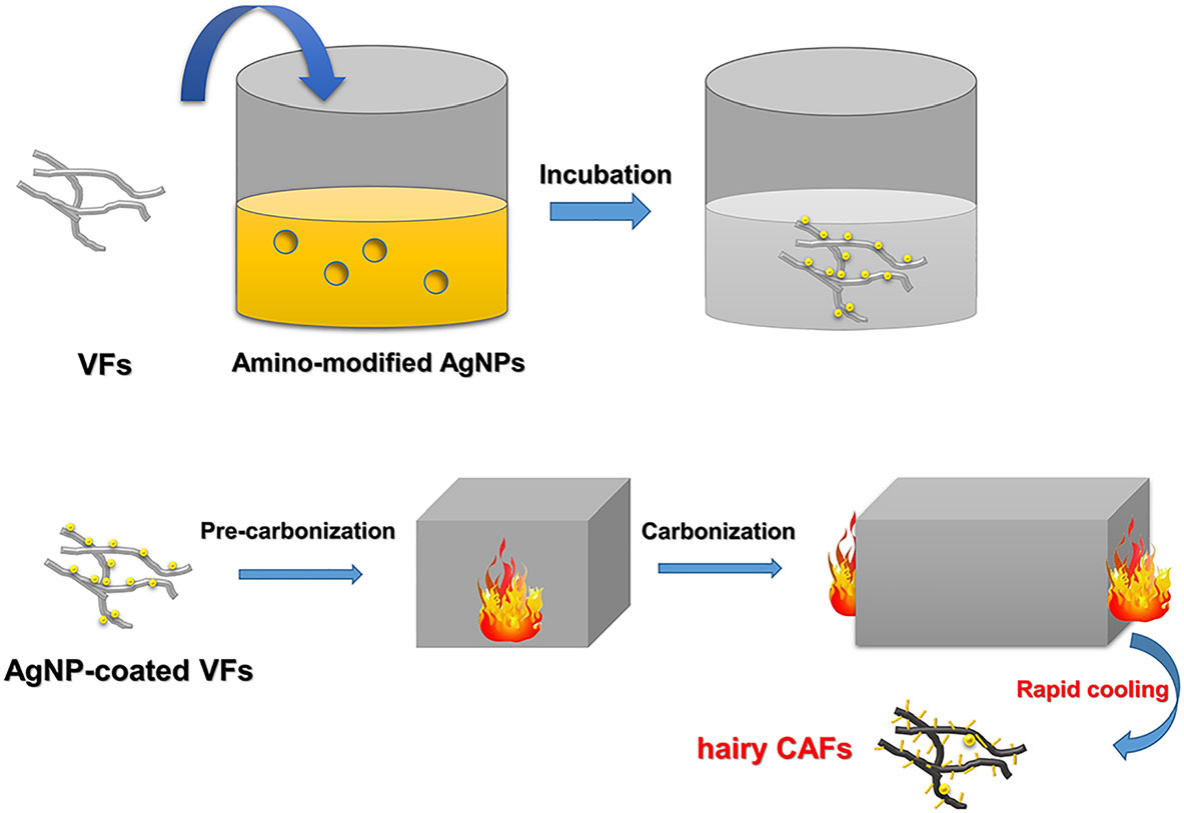
Preparation process of ACFs with dense Ag nanohair through self-assembly and thermal expansion and contraction mechanisms

Heat treatment of VFs generally entails two steps, namely, oxidation and carbonization. The precursor fiber oxidizes at 350 °C with water vapor as the activator, resulting in the formation of ladder-type polymers and allowing further processing at higher temperatures. After oxidation, the fibers carbonized at temperatures up to 850 °C under an inert atmosphere to obtain a turbostatic carbon structure. The entire procedure will be outlined in the details below. Unlike the traditional furnace, the oxidizing furnace in this study was open-ended and sealed by high-temperature flames in the entrance and exit as shown in Fig. 1. Therefore, ACFs can quickly cool down upon leaving the furnace. Note that the fast cooling process was notably vital for Ag nanohair formation.

### Measurements

Samples were characterized by FESEM (S-4200; Hitachi, Japan) equipped with an energy-dispersive X-ray spectroscopy (EDS), XPS (ESCALAB 250 XI; Thermos Scientific, USA), XRD (D8 ADVANCE, Bruker, Germany), and TG (TG 209 F3 Tarsus; Germany Netzsch Instruments, Inc., Germany). Antimicrobial activities of fiber samples were measured against *Escherichia coli* and *Staphylococcus aureus* using a shake-flask method (GB/T 20944.3-2008 [China]).

## Results and Discussion

**Fig. 2:**
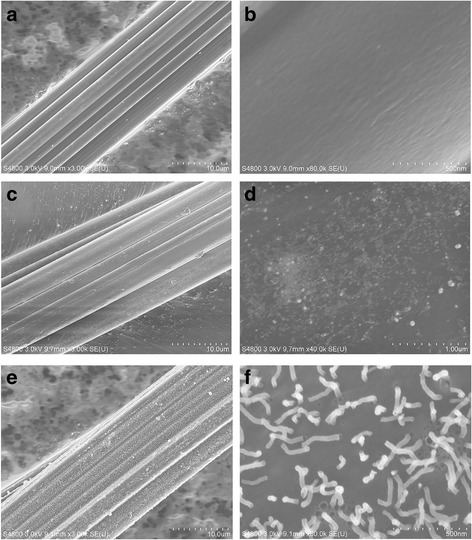
FESEM photographs of **a** × 3000 and **b** × 80,000 pure VFs, **c** × 3000 and **d** × 40,000 HBPAA/AgNP-coated VFs, and **e** × 3000 and **f** × 80,000 Ag nanohair-grown ACFs

Activated carbon fibers “thickly overgrown” by Ag nanohair were prepared through self-assembly and rapid thermal annealing as shown in Fig. 1. The surface structures of VFs, AgNP-coated VFs, and Ag nanohair-grown ACFs were first studied by FESEM (Fig. 2). Before the AgNP self-assembled on the fiber surface, VFs displayed straight longitudinal grooves along the axial direction and a clean and smooth surface in the nanoscale as shown in Fig. 2a, b. In contrast, bright white NPs with particle sizes ranging 3–80 nm occupied the surface of AgNP-coated VFs, conforming to the morphological characteristics of single AgNPs, while the longitudinal configuration remained the same. These AgNPs were monodispersed on the fiber surface, mainly attributing to the strong electrostatic repulsion among NPs. Such character may reduce the probability of AgNP self-condensation during subsequent treatment. After pre-oxidation and carbonization, fuzzy ACFs were obtained as shown in Fig. 2e. Under higher magnifications of the surface image, we found many irregularly shaped nanowires standing on the ACF surface. The particle size of the nanowires was around 50 nm, which differed from HBPPAA-capped AgNPs.

**Fig. 3:**
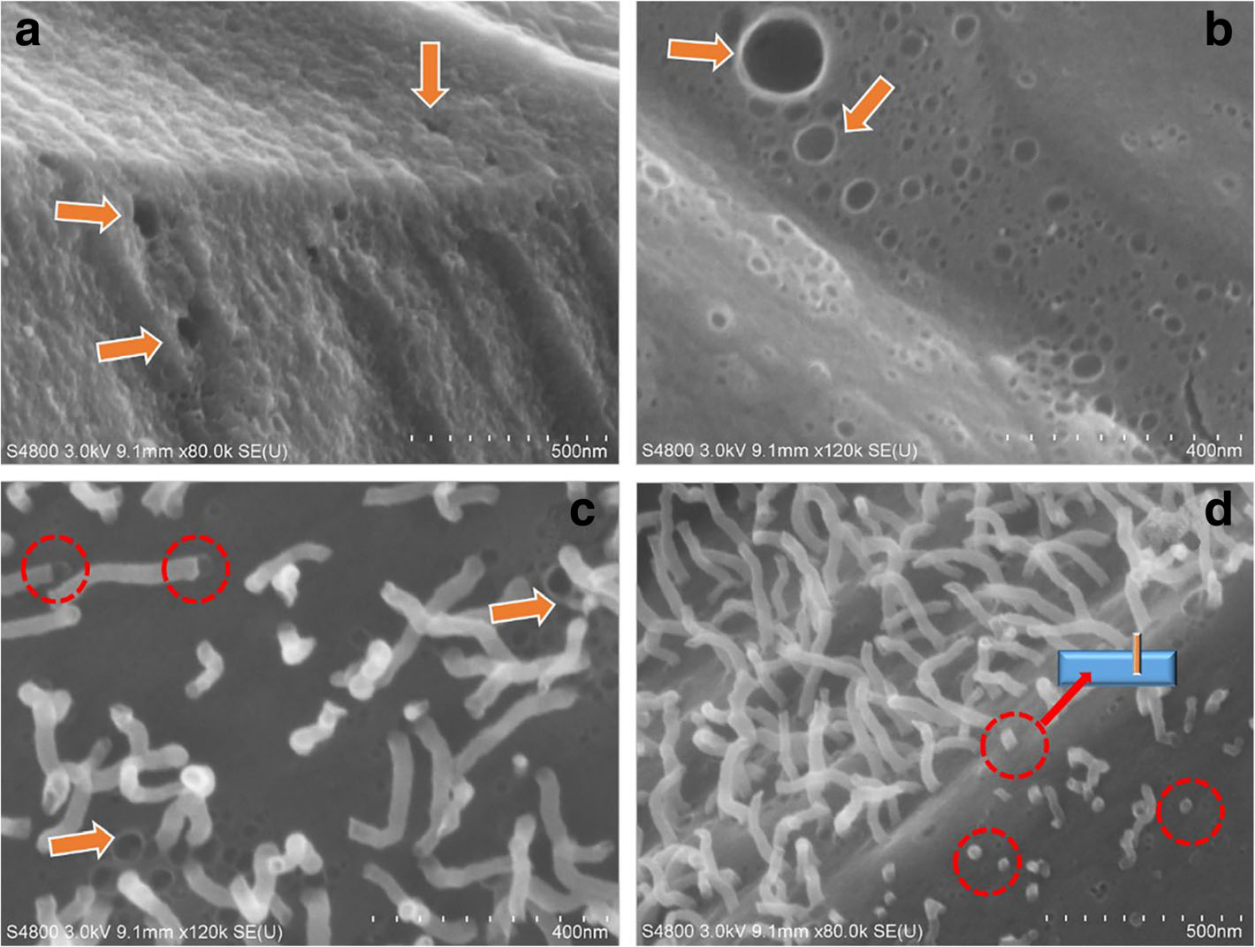
FESEM image**s** of the cross-section of the VF (**a** × 80 k) and the surface of the pure ACF (**b** × 120 k) and Ag nanohair-grown ACF (**c** × 120 k, **d** × 120 k)

To understand the possible formation mechanisms, the cross-section of VFs and the surface of pure ACFs and Ag nanohair-grown ACFs were further observed by FESEM under high resolution (Fig. 3). Many holes were present in VFs, pure ACFs, and Ag nanohair-grown ACFs, suggesting that these pores were the natural characteristics of ACFs. In addition, Ag nanowires seemed to drill the ACF surface, turning into regularly standing “hair,” as shown in Fig. 3c, d. All Ag hairs at various lengths were standing on the fiber surface. In particular, one end of Ag nanohair toppled on ACF pores, as indicated by the circular mark in Fig. 3c. The diameter of the Ag nanohair was exactly the same to the hole diameter, indicating that Ag nanohair probably grew from ACF holes because ACFs had a high porosity. Ten to 20 nm AgNPs have a very low melting point at around 129 °C. Therefore, AgNPs could be liquefied and probably adsorbed into the ACF pores through capillary effect at 850 °C [11]. When ACFs rapidly cooled down in the air, such “Ag toothpaste” could squeeze through the ACF pores, which may explain why AgNPs could form large Ag nanowires and stood on the ACF surface.

**Fig. 4:**
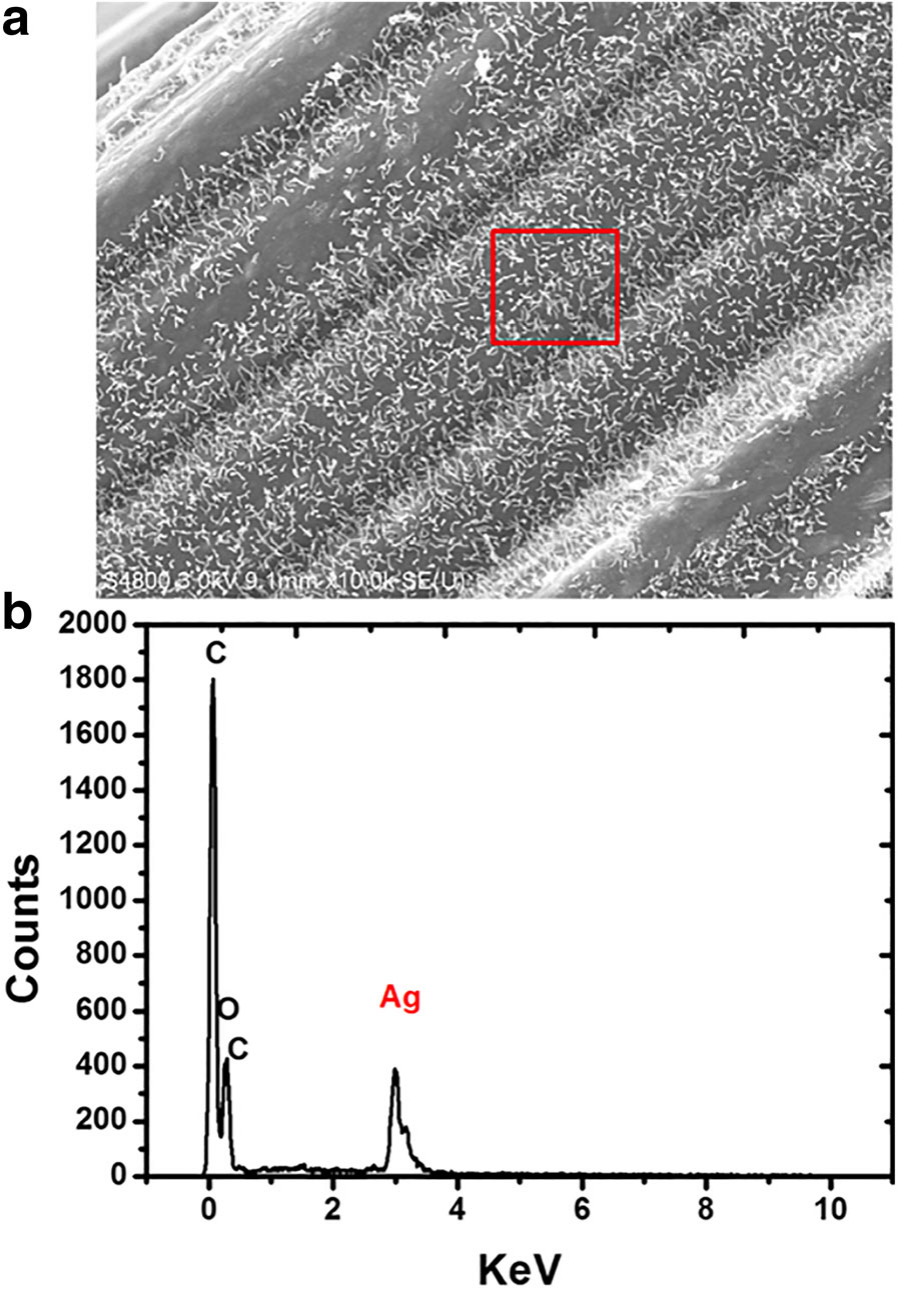
**a** SEM image and **b** EDS of the Ag nanohair-grown ACF

To verify if the Ag nanohair on the ACFs were indeed Ag, elemental composition analysis was carried out by energy-dispersive X-ray spectroscopy (EDS). The resulting EDS spectrum showed strong carbon and oxygen peaks arising from ACFs as expected (Fig. 4) [12]. The peaks of Ag in the spectrum indicated the existence of Ag in the fiber.

**Fig. 5:**
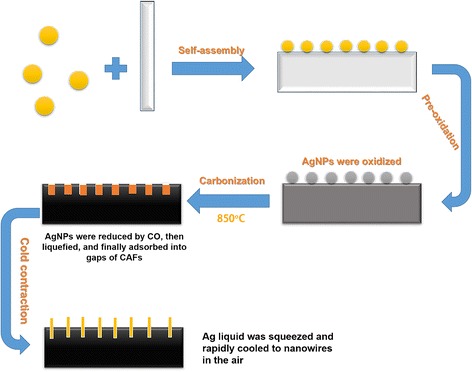
Schematic diagram for fabrication of Ag nanohair-grown ACFs

According to the analyses above, the possible mechanism may be described as shown in Fig. 5. HBPAA caps possessed abundant amino groups and positive charges and had a strong ability to self-assemble in viscose cellulose, which is mainly attributed to strong intermolecular interactions between HBPAA and cellulose. Strong repulsive interaction between AgNPs also causes monodispersion on the fiber surface. After pre-oxidation, the AgNPs on the fiber surfaces corroded to AgO or AgCl under exposure to air [13]. Nevertheless, the corrosion products can reduce to metallic Ag during subsequent high-temperature carbonization because viscose cellulose can release CO and other gaseous reductants in an oxygen-free environment [14]. Notably, solid-state AgNPs can liquefy during carbonization (the melting point of AgNPs was about 129 °C) [11]. The pores on the ACF surface adsorb the generated Ag liquid by capillary effect. When the ACFs left the furnace, these pores rapidly contracted under room temperature, spraying liquid Ag to the air and rapidly cooling down to form irregular nanowires. Because these Ag nanowires were inserted into ACFs, their rapid binding velocities may be enhanced.

**Fig. 6:**
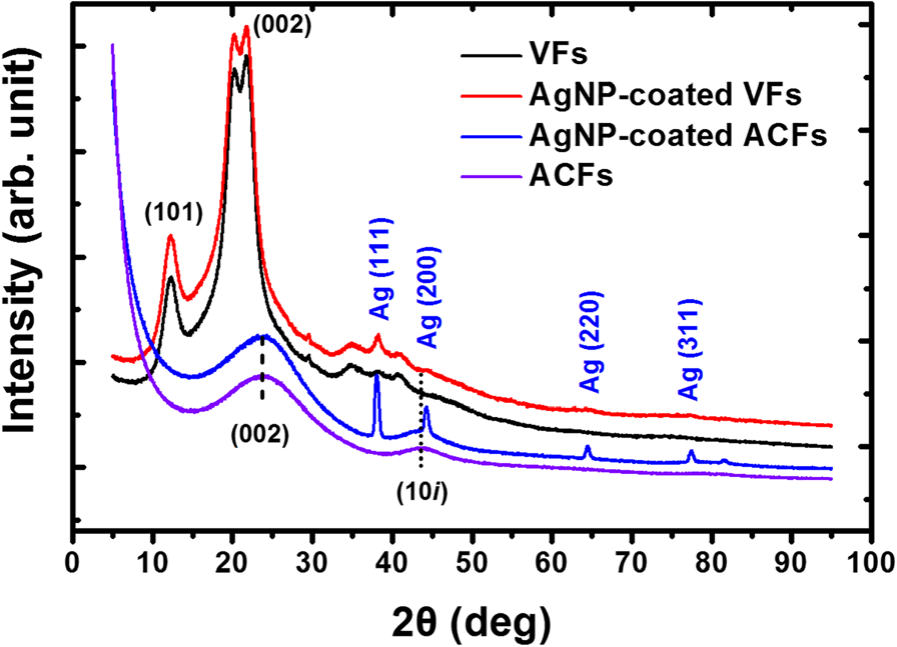
XRD patterns of (black) pure CFs, (red) AgNP-coated CFs, (blue) Ag nanohair-grown ACFs, and (purple) pure ACFs

**Fig. 7:**
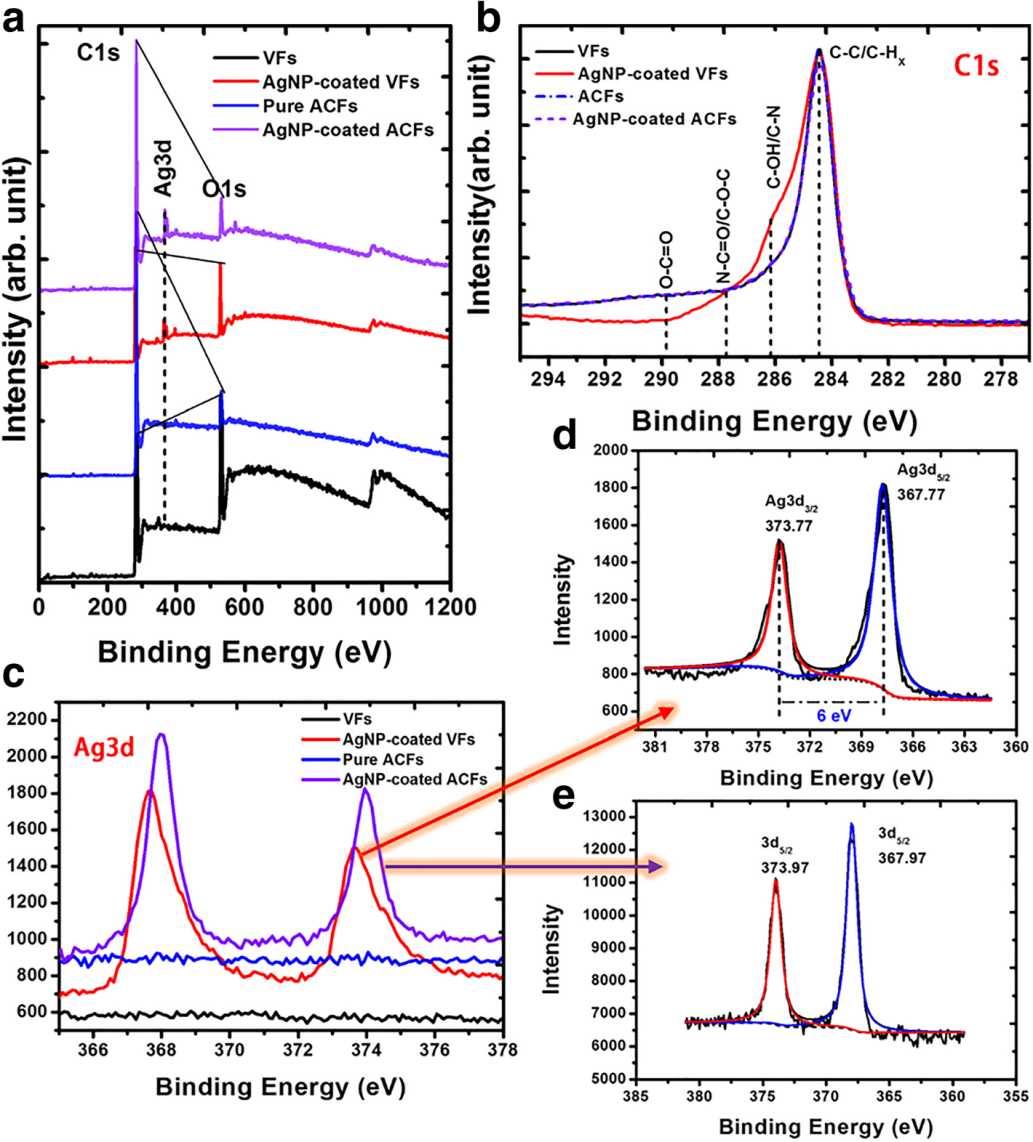
XPS spectra: **a** wide scan, **b** C1s, and **c** Ag3d spectra of pure CFs, AgNP-coated CFs, Ag nanohair-grown ACFs, and pure ACFs. **d**, **e** are Ag3d spectra of AgNP-coated VFs (**d**) and AgNP-coated ACFs (**e**)

The crystal structure and surface chemistry of the fibers were investigated by XRD and XPS (Figs. 6 and 7). As mentioned above, AgNP-coated VFs should be pre-oxidized and subsequently carbonized to generate carbon fibers. Therefore, the possible valence transition of AgNPs should be given close attention. As shown in Fig. 6, the VFs and AgNP-coated VFs had sharp peaks at around 12.3° and 21°, attributing to cellulose crystal (101 and 002 plane) [14]. By contrast, the pure ACFs and AgNP-coated ACFs had two broad peaks at around 23.5° and 43.6°, which were assigned to the disordered graphitic 002 plane and 10 plane, respectively. This turbostratic structure suggested that ACFs were composed of graphite-like microcrystalites [14]. Notably, the peak of 002 plane moves to a much higher angle (23.5°) and the peak ascribed to 10 plane appeared after the carbonization treatment, suggesting VFs were graphitized.

In addition, XRD pattern of AgNP-coated VFs showed one additional diffraction peak at around 38.3°, which can be indexed to the (111) plane of the face-centered-cubic phase of metallic Ag (JCPDS No. 04-0783) [15]. By contrast, the XRD pattern of Ag nanohair-grown ACFs showed four clear diffraction peaks at around 38.3°, 44.3°, 64.4°, and 74.5°, which can be indexed to (111), (200), (220), and (311) planes of the face-centered-cubic phase of metallic Ag (JCPDS No. 04-0783), respectively, suggesting the metal valence of AgNPs [15]. A stronger signal strength arose from the mass loss of VFs during carbonization, which also suggested that AgNPs underwent reduction during carbonization, mainly owing to the CO gas reductant generated through VF pyrolysis. In addition, the crystal structure of Ag nanohair-grown ACFs was similar to pure ACFs, indicating that Ag did not change the crystal structure.

Possible chemical change in the surface was evaluated by XPS (Fig. 7). All wide-scan XPS spectra (Fig. 7a) showed two ultra-strong peaks located at around 284 and 532 eV, corresponding to C_1s_ and O_1s_, respectively [16, 17]. These peaks mainly derived from VFs or ACFs. However, we found that the C/O ratio decreased after AgNP self-assembly, suggesting the attachment of carbonyl-containing HBPAA on the VF surface. Notably, pure ACFs and Ag hair-grown ACFs showed much higher C/O ratio, indicating the removal of most oxygen-containing groups from ACFs. Such decomposition groups probably transformed into gaseous reductants, such as CO and CH_4_, which had an ability to reduce the oxidized AgNPs to metallic AgNPs.

HBPAA was very important for self-assembly of AgNPs on VFs because it endowed AgNP surfaces with positive charges and abundant amino groups, making AgNPs compatible to negatively charged hydroxyl-containing viscose cellulose [8]. The attachment of HBPAA on VFs could be verified by analysis of C1s XPS spectra as shown Fig. 7b. The C1s peaks of four samples could be classified into four categories: carbon without oxygen bonds (C–C/C–H_*x*_) (284.5 eV), carbon single bond to oxygen or nitrogen (C–O/C–N) (286.4 eV), carbon with two oxygen and/or nitrogen bond (O–C–O/N–C=O) (287.8 eV), and carboxyl (O–C=O) (289.0 eV), attributed by VFs, ACFs, and/or HBPAA [18, 19]. Compared with VFs, ACFs, and AgNP-coated ACFs, AgNP-coated VFs showed much higher content of C–O/C–N and O–C–O/N–C=O. The enhanced peaks were owing to superposition of VFs and HBPAA.

The Ag3d deconvolution analysis shown in Fig. 7d demonstrated that the fitted Ag3d_3/2_ and Ag3d_5/2_ peaks were 373.77 and 367.77 eV for AgNP-coated VFs, agreeing with the standard values of metallic Ag (373.9 and 367.9 eV) [20]. This indicated AgNPs maintained their metallic nature when AgNPs were adsorbed to the viscose surface. Similarly, the deconvolved Ag3d_3/2_ and Ag3d_5/2_ peaks of AgNP-coated ACFs were 373.97 and 367.97 eV, suggesting the metallic state of AgNPs after the carbonization treatment (Fig. 7e). Note that the relative Ag_3d_ intensity of Ag hair-grown ACFs was much higher than that of AgNP-coated VFs, agreeing with the above-discussed XRD analysis (Fig. 7a).

**Fig. 8:**
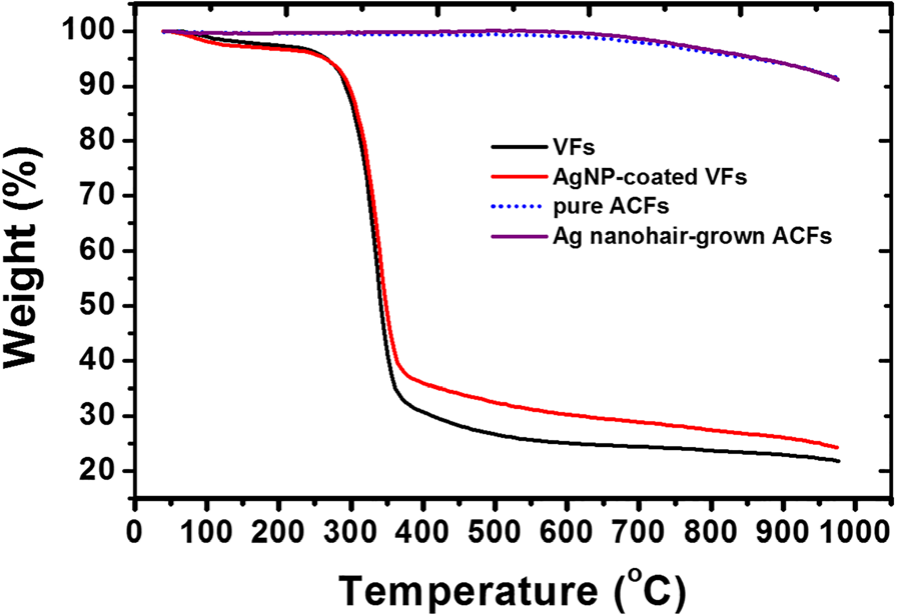
Thermogravimetric curves of (black) pure VFs, (red) AgNP-coated VFs, (blue) pure ACFs, and (purple) Ag nanohair-grown ACFs

The high thermal stability of ACFs was one of the most important characteristics. Figure 8 shows the thermogravimetric curves of VFs, AgNP-coated VFs, ACFs, and Ag nanohair-grown ACFs. Pure VFs possessed good thermal stability until 271 °C before decomposing into aliphatic char and volatile products as temperature rose from 271 to 371 °C [21]. Aliphatic char further transformed into aromatic char, generating carbon monoxide and carbon dioxide reductants, at around 485 °C [21]. For AgNP-coated VFs, HBPAA could form a hard shell over the VF surfaces and acted as a physical barrier that protected the VFs from decomposition [21, 22]. In contrast, both ACF and Ag nanohair-grown ACFs showed a weight loss of around 8.4% when the temperature reached 1000 °C, indicating excellent thermal stability and suggesting that treatment of AgNPs on fiber surfaces did not influence the thermal stability.

Antibacterial tests were finally conducted to evaluate antibacterial properties of ACFs. As shown in Table 1, ACFs showed certain antibacterial activity towards *E. coli* and *S. aureus* because the number of bacterial colonies was much lower than the original number. In contrast, the antibacterial activities of Ag nanohair-grown CFs against *E. coli* and *S. aureus* reached to almost 100 and 99.9%, respectively, demonstrating the powerful ability of AgNPs to inhibit bacterial growth [23]. After ultrasonic washing for 30 times, Ag nanohair-grown CFs still maintained excellent antibacterial properties even though its activity towards *S. aureus* slightly decreased to 97.8%. The durable antibacterial activities mainly arise from the strong adhesive strength between Ag nanohair and ACFs, which results from their interlocking structure.

**Table 1:** Antibacterial activities of ACFs against *E. coli* and *S. aureus*

Samples	Antibacterial activities			
	*E. coli*		*S. aureus*	
	Surviving cells (cfu/mL)	% reduction	Surviving cells (cfu/mL)	% reduction
ACFs	3.57 × 10^4^	–	5.4 × 10^3^	–
Ag nanohair-grown ACFs	27	~ 100	49	99.9
Ag nanohair-grown ACFs with ultrasonic washing 30 times	0	100	120	97.8

## Conclusions

Ag nanohair-grown ACFs were prepared through self-assembly of AgNPs on VF surfaces and subsequent pre-oxidation and carbonization. HBPAA served as a “molecular glue” in adhering AgNPs to VF surfaces and in forming a monodispersive AgNP coating. The Ag nanohair-grown ACFs were prepared by sequential pre-oxidation and carbonization. The growth mechanism for Ag nanohair boils down to capillary and thermal expansion effects. To instantly reduce the temperature of ACFs, we designed an open-ended carbide furnace. ACFs are immediately transferrable to the outside of the furnace after completion of carbonization. Through thermal contraction, the Ag liquid squeezed out to form Ag nanowires. Ag nanohair stood on the ACF surface and grew from the ACF pores, as shown by FESEM. XPS and XRD characterization showed that Ag had successfully self-assembled to fiber surfaces and retained their metallic state even after high-temperature carbonization, owing to the gaseous reductants generated during carbonization. TG analysis suggested that Ag nanohair-grown ACFs maintained their excellent thermal stability. Finally, the fabricated ACFs showed excellent and durable antibacterial activities as a result of their strong binding.
